# The efficacy of lavender oil on fatigue and sleep quality in patients with hematological malignancy receiving chemotherapy: a single-blind randomized controlled trial

**DOI:** 10.1007/s00520-024-09143-5

**Published:** 2025-01-08

**Authors:** Dilek Yildirim, Merve Harman Ozdogan, Seckin Erdal, Sevinc Selcuk, Azize Guneri, Elif Begum Simsek, Taha Berkay Can, Hazal Gunduz, Aysem Kuni

**Affiliations:** 1 Department of Nursing, Faculty of Health Sciences, Istanbul Aydin University, Florya Campus (Halit Aydın Campus) Inönü Street No: 38 Sefaköy – Kucukcekmece, Istanbul, Turkey; 2 Department of Medical Services and Techniques, Vocational School of Health Services, Sinop University, Sinop, Turkey; 3 Adult Hematology Unit, Acibadem Altunizade Hospital, Istanbul, Turkey

**Keywords:** Sleep quality, Sleep disorders, Aromatherapy, Hematological malignancies, Fatigue, Chemotherapy, Lavender oil

## Abstract

**Purpose:**

The aim of this study is to evaluate how aromatherapy with the inhalation of lavender oil affects fatigue and sleep quality in patients with hematological malignancies undergoing chemotherapy.

**Methods:**

This randomized, parallel-group study was carried out in the Adult Bone Marrow Transplant unit and Hematology-Oncology clinics between January 2022 and April 2023. A total of 120 patients were assigned to experimental and control groups by randomization. The study was completed with 100 patients including 50 in the experimental group and 50 in the control group. Lavender essential oil was applied to the experimental group for 20 min prior to going to bed every night for 5 consecutive days. Physiological saline solution was applied to the control group in the same way. A Participant Information Form, the Richards-Campbell Sleep Questionnaire, and the Piper Fatigue Scale were used as data collection tools.

**Results:**

The experimental group showed a significantly higher sleep quality (*p* = 0.001) and had a significantly lower PFS scores (*p* = 0.001) compared to the control group. Also, the mean scores of the experimental group on the Behavioral, Sensory, and Cognitive subscales were statistically significantly lower than the scores of the control group (*p* < 0.05). Variables of lavender aromatherapy and total sleep quality accounted for 17.1% of the variance in fatigue levels (*R*^2^ = 0.171).

**Conclusions:**

Consequently, it was determined that aromatherapy with lavender essential oil significantly alleviated fatigue and lowered PFS total and subscale scores in patients with hematological malignancies undergoing chemotherapy. Also, sleep quality significantly enhanced in the overall PFS and its subscales.

Trial registration.

NCT05808296. Date of Registration: 30 March 2023.

## Introduction

Hematological malignancies manifest themselves with the involvement in blood, bone marrow, and lymphatic system [1, 2]. According to GLOBOCAN 2020 cancer data, hematological cancers account for 6.6% of all newly diagnosed cancer cases [3].

Patients with hematological malignancies also suffer from physical and psychological difficulties due to the progression of illness and the side effects of its treatment. Chemotherapy, the primary cancer treatment method, involves high dosages of toxic agents, resulting in numerous unpleasant symptoms [4, 5]. Patients with hematological malignancies frequently experience fatigue, loss of appetite, pain, sleeplessness, anxiety, and depression induced by chemotherapy, leading to an impairment in their quality of life (QoL). A previous study investigating the QoL of cancer patients undergoing chemotherapy reported that the most common symptoms seen in these patients were fatigue, loss of appetite, sleep disturbance, and pain [5, 6].

Sleep problems are almost three times [7] more common in cancer patients than the general population and may appear before, during, and after treatment [7, 8]. Patients with hematological cancer have also sleep problems as a result of fatigue associated with the nature of cancer and its treatment process [8]. Sleep disorders may exacerbate functional impairment, fatigue, depression, and pain and pose a potential threat to immunological function. A study including acute leukemia patients demonstrated that sleep problems were one of the five symptoms that affected these patients [9, 10]. It is very important to address sleep disorders in cancer patients in order to improve their life expectancy, response to treatment and QoL, and reduce complications. Interestingly, the related studies have indicated that individuals having sleep problems are less likely to survive [11, 12] and have a lower QoL [9]. Both pharmacological and non-pharmacological methods can be used to manage sleeplessness and fatigue. Non-pharmacological approaches are also used due to the side effects of pharmacological ones and the lack of full evidence for their effects on sleep and fatigue. One of these approaches employed is the inhalation of lavender essential oil [13, 14].

Lavender aromatherapy is a well-known complementary and integrative approach. Lavender can be used since it has soothing and relaxing qualities and has no reported contraindications or side effects. It can be inhaled, ingested, or applied via transdermal patches. In general, lavender aromatherapy is considered safe as it has a very low risk for toxicity. Sedative, antidepressant, and antiemetic qualities have been reported for this aromatherapy [13–15]. The American Pharmacists Association also reports that aromatherapy with the inhalation of lavender essential oil is well tolerated in most patients and has no reported contraindication or drug interaction [13, 16].

The genus *Lavandula*, belonging to the family Lamiaceae, originally comes from the southern Europe and the Mediterranean region. Lavender has been used in traditional medicine for a long time, and the essential oil derived from lavender has a wide range of biological effects. Three lavender species are primarily selected to synthesize essential oils, including *Lavandula angustifolia* (common lavender), *Lavandula latifolia* (spike lavender), and *Lavandula intermedia*, a sterile hybrid of *Lavandula angustifolia* and *Lavandula latifolia* (lavandin). Lavender essential oil contains more than 50 chemical components that have multiple therapeutic effects. It has been used in different ways for therapeutic purposes for centuries [17, 18]. The therapeutic effects of lavender may be associated with the neurochemical effect of its volatile compounds on the limbic system in the brain and the psychological effects of its scent [17]. The use of lavender essential oil for therapeutic purposes has gradually increased, and evidence shows that lavender has antioxidant, antibacterial, antifungal, carminative, cytotoxic, antianxiety, antidepressant, and sedative properties. It is effective in alleviating migraine and reducing insomnia [17–22]. Lavender has also been used to reduce cancer complications and relieve related symptoms in patients [17, 18, 23].

Although there are studies showing the positive effect of lavender aromatherapy on the management of symptoms such as fatigue, anxiety, and insomnia [13–15, 17–22], no study results addressing fatigue and sleep quality in the same study were found in patients with hematological malignancies receiving chemotherapy. Considering that fatigue and sleep quality are two important factors that affect each other for cancer patients, these two parameters should be evaluated together. Therefore, the study aimed to evaluate how aromatherapy with the inhalation of lavender oil affected fatigue and quality of sleep in patients with hematological malignancies receiving chemotherapy.

The hypotheses of the study were the following:

H1a: The inhalation of lavender oil affects sleep quality in patients with hematological malignancies undergoing chemotherapy.

H1b: The inhalation of lavender oil affects fatigue level in patients with hematological malignancies undergoing chemotherapy.

## Methods

### Study design and setting

The study was carried out as a parallel, single-blind, randomized controlled trial with prospective, pre-test, post-test experimental design with patients with hematological malignancies who were receiving treatment in the Adult Bone Marrow Transplant unit and Hematology-Oncology clinic of a private hospital in Istanbul, Türkiye, under the guidelines of “Consolidated Standards of Reporting Trials (CONSORT)” Checklist. The clinical trial number for this study is “NCT05808296.”

This study was conducted with patients with hematological malignancies who were treated in the Adult Bone Marrow Transplant unit and Hematology-Oncology clinic of a hospital between January 2022 and April 2023 in Istanbul, Türkiye. The related clinic has a capacity of 24 beds. A total of 24 nurses were working in the unit, including 1 charge nurse, 2 clinical training nurses, 5 team leaders, and 16 staff nurses. All rooms of the clinic are single standard patient rooms. In our unit, both hematological-oncology patients and adult hematology patients who have undergone bone marrow transplantation are treated.

### Participants and sample size

The inclusion criteria for the participants were determined as (1) being 18 years old or older and agreeing to participate, (2) not having any psychiatric diagnoses, (3) not having any communication problem, and (4) having been hospitalized in hematology clinics for at least 6 days. The exclusion criteria were determined as (1) being diagnosed with a known psychiatric illness (anxiety, panic attack, depression), (2) having been a known history of allergy to lavender oil and anxiolytic drug use, (3) having arrhythmia, (3) using sleeping pills, (4) having allergy to any essential oil, and (5) using any non-pharmacological approach (warm foot baths, foot massages, listening to music, muscle relaxation) for management of fatigue and sleep-related symptoms.

A priori power analysis was performed using the G*Power 3.1.9.7 program to determine the sample size needed in the study [24]. The sample size was determined as 50 people for each group at the medium effect level (*d* = 0.5), margin of error of 5% (*a* = 0.05), and power of 80% (1-B) for the *t*-test to compare the means of independent groups.

### Blinding

The participants were blinded to grouping. Experimental and control groups received aromatherapy at different times and in separate rooms. One patient was selected per shift to prevent transmission of lavender scent between groups. While lavender aromatherapy was applied to the experimental group, colorless and odorless saline solution was applied to the control group with the same method. Moreover, the researcher who entered the data into the software and analyzed it was blind to group allocation.

### Data collection

All researchers except for the first and second researchers took part in data collection. All data were collected face to face with patients. The researchers delivered the forms to the patients, and they asked them to fill them out by giving sufficient time. The researchers collected back the completed forms. The fatigue and sleep quality of both groups of patients were examined at two time points: (1) before lavender oil aromatherapy after admission and (2) the next day (on the sixth day) after aromatherapy (for 5 consecutive days). The control group was examined at the same time points. The fatigue and sleep quality were examined using the Piper Fatigue Scale (PFS) and the Richards-Campbell Sleep Questionnaire, respectively.

### Instruments

The researchers used a Participant Information Form, the Richards-Campbell Sleep Questionnaire (RCSQ), and the Piper Fatigue Scale (PFS) to collect the data.

#### Participant information form

It was prepared by the researchers according to the literature and includes questions about the participants’ age, gender, marital status, educational background, diagnosis, and treatment type [13–15].

#### Richards-Campbell Sleep Questionnaire (RCSQ)

The RCSQ was developed Richards in 1987 [25]. The scale has six items that assess “the depth of the patients’ nocturnal sleep, the time to fall asleep, the sleep quality, the time to stay awake when awakened, the noise level in the environment, and the frequency of wake-ups.” Each item is rated on a visual analog scale. VAS ranges from 0 to 100. A score between “0–25” points indicates that the quality of the patient’s nocturnal sleep is very poor; a score between 26–75 indicates that the quality of nocturnal sleep is moderate; and a score between “76–100” indicates that the quality of nocturnal sleep is excellent. The Cronbach’s alpha coefficient was 0.82 in the validity-reliability study of RCSQ by Richards. Karaman Özlü and Özer [26] conducted its Turkish version validity and reliability study in 2015 and reported that its Cronbach’s alpha coefficient was 0.91. Its Cronbach’s alpha coefficient was found to be 0.891 in the current study.

#### Piper Fatigue Scale (PFS)

Piper B. F. et al. [27] developed the scale in 1998. Can conducted its Turkish validity and reliability study in 2001 [28]. The scale evaluates the patient’s subjective perception of fatigue. It consists of a total of 22 items and four subscales; “the behavioral/severity subscale (6 items; 2–7), which assesses the impact and level of fatigue on daily activities of life (ADL); the affection subscale (5 items; 8–12), which includes the emotional significance attributed to fatigue; the sensory subscale (5 items; 13–17), which represents the mental, physical, and emotional symptoms of fatigue; and the cognitive/mood subscale (6 items; 18–23), which reflects the level of fatigue that affects cognitive functions and mental state.” The scale also includes 5 items (1 and 24–27), which were not used to calculate the fatigue score but were recommended to remain in the scale because of their importance in the analysis of fatigue. Item 1 evaluates the duration of fatigue, while items 24–27 allow patients to express their thoughts about fatigue. Its Cronbach’s alpha value was found to be 0.946 in the current study.

### Procedures

Before the study, the patients who met the inclusion criteria were randomly assigned into experimental or control groups. Software-generated numbers (http://www.randomization.com/) were used through simple randomization (ratio 1:1). An independent person who was not involved in the study performed the randomization. Then, simple random sampling method was applied.

Afterwards, the patients were informed about the study and their consent was obtained. The Participant Information Form, the RCSQ, and the PFS were used at the beginning of the study. The researchers purchased lavender oil (contains *Lavandula hybrida*—a hybrid of *Lavandula angustifolia* and *Lavandula latifolia*) from an herbal product company registered with the Ministry of Food, Agriculture and Livestock and holding ISO22000:2005 quality certificates from the International Organization for Standardization.

Aromatherapy with the inhalation of lavender oil was applied to the patients in the experimental group, and physiological saline solution was applied to the control group using the same method. Lavender essential oil was applied to the experimental group for 20 min before going to bed every night for 5 consecutive days. It was used by dripping two drops on the sponge. The sponge was put on the patient’s shoulder (on the clothing) and fixed there. The same procedure was carried out for the control group by dripping physiological saline solution. On the sixth day, sleep and fatigue were assessed using RCSQ and PFS. Aromatherapy application was carried out by the first two researchers and the other researchers who are primary care nurses. To ensure standardization before collecting the research data, all researchers were informed about the research procedure, and their questions were answered by the first author.

### Statistical analysis

The Statistical Package for Social Science statistical software was used to analyze the data. Shapiro–Wilk and Kolmogorov–Smirnov tests were used to determine whether or not the data were normally distributed. The chi-square and independent samples *t*-test analyses were used to assess the mean, standard deviation, and difference between independent groups. The dependent groups paired sample *t*-test was used to determine the intra-group differences. All results were considered as significant at the level of *p* < 0.05 and a confidence interval of 95%. The prediction of fatigue levels of patients based on variables of lavender aromatherapy and RCSQ was assessed by multiple regression analyses. The decision about inclusion of the variables in the model was made according to the multicollinearity test. VIF and tolerance were used to assess whether or not there was multicollinearity between the variables to be included in the model. Regression analyses included variables with a tolerance value greater than 0.2 and a VIF value less than 10. No multicollinearity was found between the variables. Since no multiple linear connections were found between the variables, both lavender aromatherapy and RCSQ were included in the multiple regression analysis. Another researcher blinded to group assignment obtained all measurement scores.

### Ethical considerations

Ethical approval was obtained from Sinop University Ethics Committee (no. 2021/72, 27.05.2021) for the study. In addition, the informed consent of the participants was obtained in writing form. The Declaration of Helsinki was adhered to at every stage of the study.

## Results

**Fig. 1:**
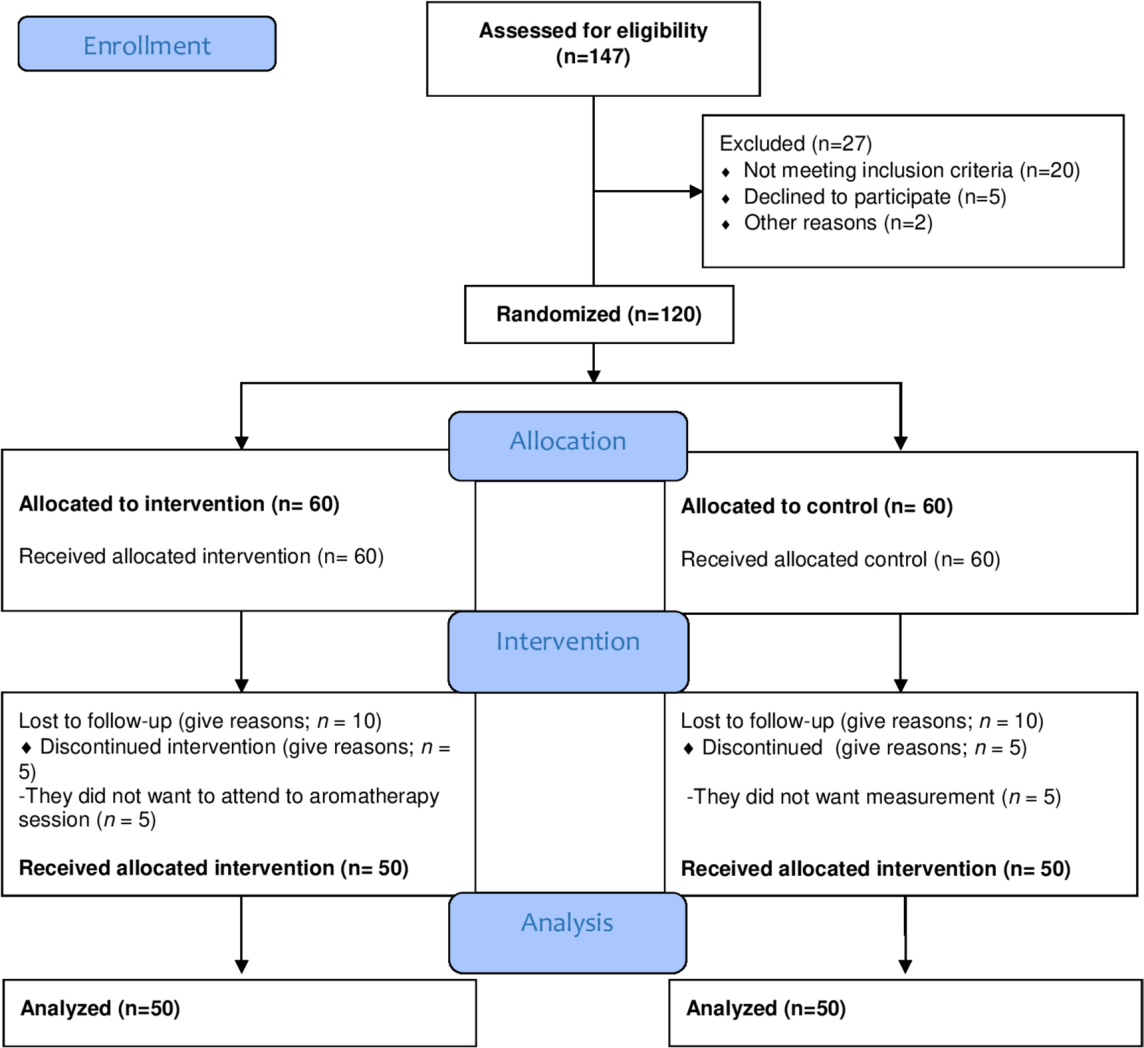
Recruitment flow diagram

The participants were assigned to the randomization program with the URL address http://www.randomization.com/. The numbers from 1 to 120 were randomly assigned to the two groups, assuming that group 1 would represent the experimental group and group 2 would represent the control group, to ensure the distribution of numbers between the groups. The order of the participants was determined by randomization in accordance with the program. Randomization was performed on 120 patients. These patients were divided into two groups and ten participants from each group withdrew due to various reasons. The study included the data of a total of 100 patients: 50 in each of the groups (experimental and control). Finally, the results of a total of 100 patients were analyzed (Fig. 1).

Table 1 shows the socio-demographic and disease-related characteristics of the participants. When the mean ages of the experimental (52.88 ± 15.87) and control (53.46 ± 14.50) groups were analyzed, no significant difference was observed between them (*p* > 0.05). When the distributions of marital status, gender, educational background, age, and employment status of the patients were analyzed based on the groups, no statistical difference was detected between the two groups (*p* > 0.05, Table 1).

**Table 1:** Descriptive characteristics of study groups (*n* = 100)

	Experimental group (*n* = 50)		Control group (*n* = 50)		Test value	
Characteristics	Mean ± SD		Mean ± SD		*t*	*p*
Age	52.88 ± 15.87		53.46 ± 14.50		− 0.191	0.849
	*n*	%	*n*	%	χ^2^	*p*
Gender						
Female	16	32	18	36	0.178	0.417
Male	34	68	32	64		
Marital status						
Single	12	24	13	26	0.053	1.000
Married	38	76	37	74		
Working status						
Yes	22	44	27	54	0.640	0.424
No	28	56	23	46		
Education level					2.454	0.293
Primary	6	12	10	20		
High school	16	32	10	20		
University	28	56	30	60		
Smoking cigarettes					10.485	0.005
Yes	8	16	4	8		
No	36	72	26	52		
Quit	6	12	20	40		
Night sleep pattern						0.840
Yes	28	56	30	60	0.164	
No	22	44	20	40		
Night sleep time					2.870	0.238
Less than 5 h	15	30	9	18		
5–8 h	29	58	37	74		
More than 8 h	6	12	4	8		
Diagnosis						
Multiple myeloma	18	36	18	36		
ALL	10	20	3	6		
AML	7	14	10	20	7.828	0.251
KLL	1	2	3	6		
Non-Hodgkin lymphoma	6	12	3	6		
KML	1	2	3	6		
Hodgkin lymphoma	7	14	10	20		
Number of chemotherapy cycles						
First cycle	41	82	33	66		
Second cycles	6	12	4	8	11.932	0.217
Fourth cycles	-	-	8	16		
Fifth cycles	2	4	4	8		
Sixth cycles	1	2	1	2		
Unit receiving treatment						
The bone marrow transplant unit	11	22	9	18	0.250	0.803
Hematology-oncology clinic	39	78	41	82		

χ^**2**^**,** chi-square test was used. *p* < 0.05. *SD* standard deviation. *t* independent *t*-test

Nocturnal sleep patterns, daily sleep duration, diagnosis, and chemotherapy treatment cycles of the experimental and control groups were examined, and no statistically significant difference was found between them (*p* > 0.05). The two groups were similarly or homogeneously distributed in terms of all these characteristics (Table 1).

Before the intervention (lavender aromatherapy application), sleep depth (deep-light sleep), waking frequency, time to fall asleep, sleep quality, falling asleep when awakened, environmental noise level, and total sleep quality characteristics of the experimental and control groups were similar (*p* > 0.05). After the intervention, the waking frequency and falling asleep time of the experimental group decreased compared to the control group and also the sleep depth improved (*p* < 0.05). In addition, the RCSQ mean scores of the experimental group were statistically significantly higher than the scores of the control group after aromatherapy (70.80 ± 16.89 vs. 59.16 ± 17.83; *p* < 0.05). However, there was no statistically significant difference between the groups in terms of the RCSQ subscales and sleep quality scores after the intervention (*p* > 0.05) (Table 2).

**Table 2:** The comparison of the Richard Campbell Sleep Questionnaire scores of the groups (*n* = 100)

		Experimental group *n* = 50	Control group *n* = 50		
Pre-test	Richard Campbell Sleep Questionnaire	Mean ± SD	Mean ± SD	*t*	*p*
	Sleep depth	57.40 ± 26.60	57.10 ± 20.45	0.063	0.950
	Falling asleep	56.60 ± 25.66	56.10 ± 25.62	0.097	0.923
	Number of awakenings	54.60 ± 28.01	54.90 ± 22.51	− 0.059	0.953
	Percentage of time awake	57.60 ± 26.24	58.10 ± 24.72	− 0.098	0.922
	Sleep quality	58.50 ± 23.65	62.80 ± 21.07	− 0.960	0.340
	Sleep index	70.80 ± 26.84	76.50 ± 23.24	− 1.135	0.259
	Richard Campbell Sleep Questionnaire Total Score	56.94 ± 22.72	57.80 ± 17.97	− 0.210	0.834
Post-test		Experimental Group *n* = 50	Control Group *n* = 50		
	Richard Campbell Sleep Questionnaire	Mean ± SD	Mean ± SD	*t*	*p*
	Sleep depth	73.00 ± 19.14	58.30 ± 19.68	3.786	** < 0.001**
	Falling asleep	71.30 ± 18.86	56.70 ± 25.50	3.193	**0.002**
	Number of awakenings	69.40 ± 20.04	56.20 ± 21.84	3.149	**0.002**
	Percentage of time awake	70.50 ± 18.13	61.90 ± 22.78	2.088	**0.039**
	Sleep quality	69.80 ± 16.90	62.70 ± 21.19	1.852	0.067
	Sleep index	78.30 ± 20.16	76.60 ± 22.95	0.393	0.695
	Richard Campbell Sleep Questionnaire Total Score	70.80 ± 16.89	59.16 ± 17.83	3.350	**0.001**

*t*, independent samples *t*-test. *p* < 0.05 is written in bold

Table 3 shows the PFS scores of the patients. The pre-intervention PFS mean scores of the experimental and control groups and their mean scores in the Behavioral, Affection, Sensory, and Cognitive subscales were similar to each other (*p* > 0.05). After the intervention, the PFS mean scores of the experimental group were statistically significantly lower compared to the control group (*p* < 0.05). Also, the mean scores of the experimental group on the Behavioral (4.93 ± 1.77 vs. 5.81 ± 2.27), Sensory (4.56 ± 1.74 vs. 5.85 ± 1.95), and Cognitive (4.03 ± 1.72 vs. 4.95 ± 1.60) subscales were statistically significantly lower compared to the control group (*p* < 0.05). However, there was no statistically significant difference between the groups in terms of the mean scores of the affection subscale after the intervention (*p* > 0.05). When the mean scores of PFS and its subscales were compared in the experimental group, the post-intervention mean scores were significantly lower than pre-intervention mean scores (*p* < 0.05) (Table 3).

**Table 3:** The comparison of the Piper Fatigue Scale (PFS) scores of experimental and control group

PFS Subscales			Experimental	Control		
		Groups	Mean ± SD	Mean ± SD	t*	*p*
The Piper Fatigue Scale (PFS)		Pre-test	5.70 ± 1.66	5.50 ± 1.65	0.561	0.576
		Post-test	4.54 ± 1.16	5.51 ± 1.64	− 3.394	**0.001**
		t**	*5.151*	*0.149*		
		*p*	** < *0.001***	*0.882*		
**Subscales**	Behavioural	Pre-test	5.78 ± 2.03	5.82 ± 2.32	− 0.099	0.921
		Post-test	4.93 ± 1.77	5.81 ± 2.27	− 2.147	**0.034**
		t**	*2.797*	*0.449*		
		*p*	***0.007***	*0.655*		
	Affect	Pre-test	5.97 ± 2.14	5.42 ± 2.33	1.225	0.224
		Post-test	4.65 ± 1.57	5.43 ± 2.29	− 1.963	0.052
		t**	*3.738*	*1.396*		
		*p*	** < *0.001***	*0.169*		
	Sensory	Pre-test	5.81 ± 1.58	5.97 ± 1.81	− 0.112	0.911
		Post-test	4.56 ± 1.74	5.85 ± 1.95	− 3.474	**0.001**
		t**	*4.364*	*1.567*		
		*p*	** < *0.001***	*0.124*		
	Cognition/mood	Pre-test	5.23 ± 1.92	4.97 ± 1.49	0.766	0.446
		Post-test	4.03 ± 1.72	4.95 ± 1.60	− 2.776	**0.007**
		t**	*4.030*	*0.875*		
		*p*	** < *0.001***	*0.159*		

t*, *t*-test independent groups; t**, *t*-paired samples *t*-test; *p* < 0.05 is written in bold

Multiple regression analysis was applied to predict lavender aromatherapy application and total sleep quality variables and fatigue. The results of the analysis indicated that the model constructed was statistically significant (*F* = 9.924, *p* < 0.001). Variables of lavender aromatherapy and total sleep quality included in the model were statistically significant predictors of fatigue (*p* < 0.05). Lavender aromatherapy and total sleep quality variables accounted for 17.1% of the variance in fatigue levels (*R*^2^ = 0.171) (Table 4).

**Table 4:** Evaluation of the independent variables that have an effect on the fatigue: multiple regression analysis

	B	Standard error	Standard beta (*β*)	*t*	*p*	95% CI	
Constant	6.559	0.496		13.211	< 0.001	5.573 to 7.544	
Lavender Aromatherapy	− 0.863	0.284	− 0.299	− 3.045	**0.003**		− 1.426 to − 0.301
Post-test RCSQ scores	− 0.016	0.008	− 0.205	− 2.091	**0.039**		− 0.032 to − 0.001

Dependent variable: post-test; the Piper Fatigue Scale total scores

*R* = 0.414; *R*^2^ = 0.171; adjusted *R*^2^ = 0.154; *F* = 9.924; *p* < 0.001; Durbin Watson = 1.932 (1.5–2.5), *p* < 0.05 is written in bold

## Discussion

The results of the current study revealed that aromatherapy with the inhalation of lavender essential oil improved “sleep depth, falling asleep, number of wake-ups, percentage of time awake, and total sleep quality.” Although the literature contains a considerably limited number of studies conducted with patients with hematological malignancies, lavender aromatherapy enhanced the sleep quality of patients (experimental group 70.80 vs. control group 59.16, *p* = 0.001), similar to the results of studies conducted with cancer patients. Hamzeh et al. [29] reported that cancer patients inhaled lavender and peppermint oil for 20 min at 21:00 at night for 7 days, and this significantly enhanced their sleep quality. The patients receiving palliative care took ten deep breaths over a bowl of three ml of lavender oil at 22:00 at night for 3 days, and then the bowl was left 1 m away from their bed and kept until 06:00 in the morning. As a result, lavender oil enhanced sleep quality by enhancing the depth of sleep, shortening the time to fall asleep, and reducing the frequency of wake-ups [15]. Özkaraman et al. [14] stated that the anxiety level of cancer patients on chemotherapy who smelled lavender oil for 5 min every evening for 30 days lowered and their sleep quality enhanced significantly. The blood pressure level of patients receiving aromatherapy with 3 ml of lavender oil placed in a glass jar beside their beds between 22:00 and 06:00 every night in the intermediate intensive care unit was lower, and their total sleep scores were higher [30]. The result of the present study is compatible with the results of other studies, and Hypothesis 1a was confirmed.

Another important result of this study is that the fatigue levels of patients (experimental group 4.54 vs. control group 5.51, *p* = 0.001) lowered after lavender oil aromatherapy. Aromatherapy, one of the integrative interventions, lowers the level of fatigue and alleviates its severity. Especially when applied through inhalation, aromatherapy is an easy, fast, and effective treatment method for some “physical and physiological problems such as upper and lower respiratory tract infections, fever, headache, sinusitis, fatigue, depression, and sleeplessness” [31, 32]. Previous studies indicated no results on how lavender aromatherapy affected fatigue in patients with hematological malignancies. However, there are study results on how aromatherapy with the inhalation of lavender oil affected the fatigue of patients during hemodialysis. Muz and Taşçı conducted a study to determine how aromatherapy with inhalation of orange and lavender affects sleep quality and fatigue levels in patients during hemodialysis session and reported that the aromatherapy enhanced sleep quality, lowered the level of fatigue, and alleviated its severity in these patients [32]. In their study, Bicer and Demir [31] reported that aromatherapy with the inhalation of lavender oil significantly alleviated the severity of fatigue in patients who underwent hemodialysis and determined that it had no side effects and was affordable and easy-to-apply by nurses. The result of the present study is compatible with previous studies. According to these data, Hypothesis 1b was confirmed. Additionally, no side effects of lavender oil were observed in the present study.

### Limitations

This study investigated only the short-term effects of inhalation of lavender essential oil. Therefore, its long-term effects are unknown. Another limitation is that the study was conducted with all patients with hematological malignancy, and the results were not analyzed according to disease type. Another limitation of the study is that the length of hospital stay of the patients for aromatherapy application was ignored.

## Conclusions

Consequently, this study indicated that aromatherapy with the inhalation of lavender essential oil can significantly alleviate the severity of fatigue and lower the PFS total and subscale scores in patients with hematological malignancy. Likewise, sleep quality significantly enhanced in overall PFS and its subscales. Haemato-oncology nurses should routinely assess fatigue and sleep quality. Hemato-oncology nurses can use the aromatherapy with the inhalation of lavender essential oil as an independent nursing practice. No side effects of lavender oil were observed in the study. It is affordable, safe, non-invasive, simple, and reliable for management of fatigue and sleep problems in patients with hematological malignancies.

Considering the results of present study, aromatherapy with the inhalation of lavender essential oil has positive effects on quality of sleep and fatigue in patients with hematological malignancies when administered via inhalation. However, it is recommended to carry out long-term studies with a large sample group in order to evaluate its long-term effects.

## Data Availability

No datasets were generated or analysed during the current study.
